# Postspinal Anesthesia Hypotension in Caesarean Delivery: A Narrative Review

**DOI:** 10.7759/cureus.59232

**Published:** 2024-04-28

**Authors:** Samarpan Patel, Sanjot Ninave

**Affiliations:** 1 Anesthesiology, Jawaharlal Nehru Medical College, Datta Meghe Institute of Higher Education and Research, Wardha, IND

**Keywords:** obstetric anesthesia, spinal anesthesia, postspinal hypotension, c-section, pregnancy

## Abstract

Anesthesiologists frequently deal with spinal hypotension when administering spinal anesthesia (SA) for a Caesarean section (C-section). The physiological changes that occur during pregnancy necessitate modifications to anesthesia and analgesia procedures to provide safe and efficient care for the expectant patient. It is believed that giving the patient SA during a C-section will increase their degree of comfort and pain management both during and after the surgical process. It is less expensive, easier to give, and delivers a consistent anesthetic onset, early ambulation, and the start of breastfeeding. As C-section is a very common operation performed in every healthcare unit, dealing with postspinal hypotension is a daily situation faced by anesthetists with variable levels of experience. However, understanding and addressing hypotension induced by SA is crucial as it affects the mother and the fetus negatively. This review aims to contribute to enhancing patient care and safety in the context of C-sections by identifying hypotension timely and managing it effectively. It is advised to healthcare workers to leverage the insights from the review to improve patient outcomes in routine practice.

## Introduction and background

The most common surgical technique performed in obstetrics and gynecology is the Caesarean section (C-section). Roughly 30% of live births occur via this method. Even while C-section deliveries are becoming more common worldwide and are safer than in the past, they are still linked to greater rates of maternal and neonatal death and morbidity than vaginal births. In addition to the surgical method, the anesthetic utilized is also a contributing factor in the higher rates of death and morbidity. Over time, there has been a sharp increase in C-section rates worldwide, from about 7% in 1990 to 21% now, above the WHO's recommended range of 10% to 15%. Across India, the proportion of Caesarean deliveries grew from 17.2% to 21.5% between the National Family Health Survey (NFHS)-4 (2015-16) and NFHS-5 (2019-21) [1]. When a C-section is performed, anesthesia can be either general or local. In most cases, local or regional anesthesia (RA) is used during C-section, as it is a generally safer alternative to general anesthesia (GA). Spinal anesthesia (SA), epidural anesthesia, and combination anesthesia are examples of RA [2]. Because of its simplicity, safety, ease of administration, capacity to deliver appropriate surgical anesthesia, quicker onset of action, and ability to increase mother satisfaction through early skin-to-skin contact, single-shot SA is favored for elective Caesarean delivery. In 1900, Kreis used an intrathecal cocaine injection to perform the first SA for obstetrics during an operative vaginal delivery [3]. Hopkins originally reported using a hyperbaric spinal anesthetic for C-sections in 1902 [4]. Pitkin used it for the first time for obstetric delivery in 1928 [5]. Although some still reported great results, reports of maternal mortality caused its use to decline in the 1930s [6]. However, in case the SA fully fails, the anesthesiologist must be prepared to introduce GA, despite the numerous grounds against use in pregnant females because of the heightened aspiration risk and challenging intubation procedures [7]. A recurring problem during SA for C-section is maternal hypotension. Hypotension incidence under SA differs across several studies, falling between 7.4% and 74.1%, even following preventive interventions [8]. In addition to being a significant contributing factor to maternal death associated with RA, acute SA-induced hypotension can arise suddenly and have a substantial detrimental effect on the fetus, accompanying vomiting/nausea in the mother as well as fetal acidosis. Heart failure may occur in mothers who have hypovolemia prior to birth, as the sympathetic blockade may significantly reduce venous return (VR) [9].

## Review

Anesthesia for C-section delivery

There are various indications for C-section delivery (Table 1). However, to perform a C-section, the mother and the fetus's health as well as the procedure's urgency should be taken into consideration when selecting an anesthesia. Following a thorough examination of the advantages and disadvantages of each anesthesia option, the mother's preferences must be taken into account. The mother and the newborn should have access to resuscitation equipment prior to the start of any anesthetic procedure. In obstetrics, regional approaches offer a number of benefits. They prevent the need for sedative anesthetics, lower the risk of gastric aspiration, and let the woman stay awake during delivery. When using RA instead of GA, operational blood loss may also be decreased. Due to its quick onset and consistency, subarachnoid block is perhaps the most commonly used RA during Caesarean delivery. In emergency C-sections, it has also replaced GA [10,11].

**Table 1:** Indication for C-section delivery C-section: Caesarean section

Maternal Indication	Fetal Indication	Anatomic Indication
Prior Cesarean delivery	Malpresentation	Placental abruption
Maternal request	Congenital anomaly	Placenta previa/accreta
Cephalopelvic disproportion	Umbilical cord prolapse	Invasive cervical cancer
HIV/herpes infection	Macrosomia	Genital tract obstructive mass
Cerebral aneurysm	Thrombocytopenia	Permanent cerclage

Pathophysiology of spinal hypotension

Sympatholytic Response

The primary pathophysiological pathway by which SA results in hypotension is the immediate initiation of the sympatholytic response triggered by the nerve fibers' increased sensitivity to local anesthetics during pregnancy. There is a relationship between the sympathetic chain blockage level, which is frequently erratic and multiple dermatomes above the sensory block level, often unpredictable, and the extent to which the local anesthetic inside the subarachnoid area has diffused throughout the brain [12].

Aortocaval Compression (AC)

Compared to non-obstetric patients, pregnant women experience hypotension more frequently and at higher levels. This is mostly because of the AC of the uterus during pregnancy and a greater susceptibility to local anesthetics. The expectant mother is more vulnerable to reductions in cardiac output (CO) and VR when AC is present. There is a decrease in systemic venous resistance (SVR) and a loss of sympathetic tone during SA. This drop in SVR lowers the VR even more and is linked to a drop in the mother's CO and a reflex rise in HR. Additionally, compared to parasympathetic activity, pregnant women have higher levels of sympathetic activity. Consequently, more peripheral vasodilatation and a predominance of parasympathetic activity are brought on by sympatheticolysis, which produces bradycardia, nausea, and vomiting by lowering the cardiac preload and VR [13,14].

Decreased Preload

Systemic hypotension is caused by the reduction in preload, which also lowers CO, and mostly results from sympathetic blocking, which causes peripheral vasodilatation and blood pooling in the veins. Hypotension results from a reduction in CO and VR. The condition is made worse by aortic constriction. Through baroreceptors, elevated sympathetic block inversely reduces the frequency of compensatory responses, and it increases the risk of cardioinhibitory responses such as the Bezold-Jarisch reflex, which ultimately results in cardiac arrest and death [15]. In addition to the previously mentioned mechanism, maternal body mass index (BMI) and SA method are known risk factors for hypotension development. According to certain research, people with higher BMIs may be more likely to experience hypotension when receiving SA for Caesarean delivery. According to one study, underweight growth (less than 11 kg relative to prenatal weight) increased the risk of hypotension and changed HR variability (HRV) patterns during SA [16-18].

Autonomic function assessment

The autonomic nerve system (ANS) regulates systemic hemodynamics. Determining the autonomic tone before surgery may offer a chance to identify people who may experience severe hemodynamic impairment after surgery [19]. Changes in autonomic function have been linked to a higher risk of hypotension after SA, according to several studies.

Given the ANS's crucial function in regulating blood pressure (BP) during pregnancy and the postpartum period, parturients who are more likely to have hypotension may be identified by assessing the preoperative ANS activity. Specifically, analyzing HRV is a noninvasive method to assess how active different ANS components are. The variation in the intervals between successive heartbeats is known as HRV, and it can be generally analyzed in terms of time, frequency, or nonlinear domains. Prior research on SA-induced hypotension has concentrated on analyzing the frequency domain (FD), more especially, the low-frequency to high-frequency (LF/HF) ratio, which is the ratio of HF (0.20 to 0.40 Hz) to LF (0.04 to 0.15 Hz) power. This is due to the theory that the LF/HF ratio measured the equilibrium between the ANS's sympathetic and parasympathetic components, with higher LF and HF power corresponding to higher levels of sympathetic and parasympathetic activity, respectively [20].

These investigations show that the occurrence of hypotension during SA is predicted by lower preoperative LF/HF ratios, which are indicative of decreased sympathetic activity. Preoperative lower standard deviation of the NN interval (SDNN), higher standard deviation 2 (SD2), and lowered systolic BP (SBP) were linked to hypotension during SA, according to a study by Du et al [20]. Additionally, the stronger ANS compensation for pregnancy-induced vasodilation may limit the ability of the ANS to offset further drop in SVR generated by SA [21].

According to a recent study, the ANS index and other techniques for measuring autonomic function may also be useful in anticipating obstetric spinal hypotension. Although this study was limited in comparison to the number of factors investigated, it was unable to validate the LF/HF ratio as a predictor of spinal hypotension. In addition to HRV, other markers that can be used to predict when maternal hypotension will start after C-sections performed under SA include the Pleth Variability Index, baseline HR, cerebral oxygen saturation, and pulse transit time. Measuring these variables, however, is challenging in the recovery or operating rooms right before surgery since they necessitate the use of particular software and equipment that are sometimes not available in these settings [22].

Additionally, research indicates that when diagnosing diabetic autonomic dysfunction, the autonomic function test is more sensitive and specific than vasomotor tests, such as assessing total pulse amplitude. Problems like poor temperature control and compromised stress tolerance are especially pertinent during the perioperative phase and certainly apply to C-sections [23].

Importance of timely predicting hypotension

Nausea and vomiting are significantly more likely during SA following a C-section than during non-obstetric surgery, and they are mostly brought on by hypotension. In addition to inducing temporary brainstem ischemia and activating vomiting centers, acute hypotension lowers cerebral perfusion. Studies using near-infrared spectroscopy (NIRS) have indicated that this may also lead to transitory cerebral hypoxia associated with a notable drop in the mother's cerebral blood volume, cerebral oxygen saturation, and cerebral oxygenation [24]. This is in line with research showing that consuming oxygen can reduce the risk of feeling queasy and avoid brain hypoxia. Severe and prolonged hypotension in mothers might result in vertigo and a diminished level of awareness. When the drop in BP is immediately treated, these adverse effects are less frequent. SA causes a 20% reduction in splanchnic blood flow, which is more noticeable when systemic hypotension is present [25]. Another pathologic explanation of nausea and vomiting is the release of emetogenic chemicals, such as serotonin, from the digestive system as a result of splanchnic hypoperfusion. Research on the impact of hypotension after C-sections on human fetal physiology is still lacking; however, studies on animals indicate that a previously uncompromised fetus that continues to have uteroplacental blood flow reduced by more than 60% develops bradycardia and acidemia. Babies born to moms experiencing hypotension caused by SA had considerably higher acidotic levels [26].

Spinal hypotension is frequently experienced following C-sections, and up to 80% of anesthetics must be used in combination with vasopressors to address it. As will be seen later, the precise incidence of hypotension varies greatly depending on the definition. Given these conditions, one may contend that only choosing to give an SA would be sufficient to anticipate hypotension [27,28]. Moreover, several editorials have questioned whether trying to avoid hypotension is necessary because treating it is so effective. Even though these are significant issues, most obstetric anesthetists are still very interested in the prevention and management of hypotension [29].

Moreover, there is a correlation between hypotension and a higher incidence of fetal acidemia and nausea and vomiting in parturients, as well as maternal and fetal morbidity. Predicting which patients will experience severe hypotension would also allow for appropriate preoperative planning and may lead to modifications in treatment plans, such as early vasopressor infusion in high-risk patients. Patients receiving care in a peripheral environment who are at risk of developing severe hypotension may be referred to more easily accessible high-care facilities and places with access to specialized anesthesia services [30].

Although SA-induced sympathectomy appears to be the primary cause of hypotension, it is also clear that an active ANS is necessary for the compensatory mechanisms needed to keep the BP stable. Consequently, an evaluation of the ANS may give medical professionals important knowledge about a patient's capacity to handle hemodynamic instability [31]. However, autonomic function testing is complicated and differs from bedside tests reported more than 30 years ago to the more sophisticated methods employed today. One of these techniques is measuring the HRV of patients prior to surgery, and the results are statistically analyzed [32].

Several statistical tests can be run, such as time domain, FD, and nonlinear analysis. Though now a little out of date, a task committee made an effort to offer standards for the usage of this technology, associated equipment, and statistics. The first study to show that the HRV methods might be used to anticipate spinal hypotension in individuals having an elective C-section was Chamchad et al. [32]. They assessed the potential of the HRV measure point correlation dimension (PD2) to predict hypotension that coexists with SA for Caesarean delivery. They showed that hypotension following SA for Caesarean delivery is predicted by a baseline pPD2 of >3.90, an unbiased discriminant derived from the median value. According to their findings, HRV examined using pPD2 exhibits potential as a new predictor of postspinal hypotension [33].

Compared to traditional measures like baseline HR, the PD2 performed better and effectively identified which women were likely to develop hypotension [34]. After that, Hanss et al. employed FD analysis of the HRV to forecast when spinal hypotension would occur during a Caesarean birth [34]. They did this by first identifying the at-risk group using a retrospective model and then verifying it with a prospective group. They employed an LF/HF ratio variable, which is believed to represent the balance between the sympathetic and parasympathetic nervous systems. Retrospective HRV analysis was performed on 41 patients, who were divided into three divisions based on the degree of SBP decline: mild, moderate, and severe. It was discovered that following subarachnoid block (SAB), the SBP of high-risk individuals was significantly lower (76 +/- 21 vs. 111 +/- 12 mmHg; P < 0.05). Severe hypotension was anticipated by high LF/HF before SAB. Preoperative HRV measurement may identify those who could have hypotension following SAB [35].

Research on obstetric patients indicated that individuals having spinal blocks had decreased LF/HF values, suggesting an assumed lower sympathetic outflow. They showed that patients were more prone to spinal hypotension if they had higher sympathetic tone and therefore better balance. Crucially, if a measurement of HRV was conducted the day before surgery, it was not predictive of hypotension [36].

Afterward, Hanss et al. effectively guided the preventive management in patients who were at risk of experiencing severe low BP using these indicators [36]. Subsequent research conducted outside of the obstetric context revealed that changes in HRV were predictive of hypotension during SA, as demonstrated by measurements of entropy and the LF/HF ratio [37].

Incidence and risk of hypotension

An institutional cross-sectional study involving 410 patients found that 64% of females showed that the weight of the baby, baseline SBP (BSBP), duration between skin incision and spinal induction, the height of the sensory block and the anesthetist's experience were linked to hypotension. Females whose newborns weighed more than 4 kg were five times more likely to experience hypotension than mothers whose newborns weighed less than 2.4 kg (95% CI: 1.6-17.7) [38]. Additionally, the investigators discovered that a sensory height block greater than T6 increased the incidence of hypotension by double. Hypotension is more likely to occur the longer the period of time between the skin incision and spinal induction. One plausible explanation could be that as the lengthening of time between spinal induction and skin incision, so does the amount of time expectant mothers spend in a supine position before giving birth. The danger of hypotension is increased by this increase in the AC time and the inhibition of sympathetic responses brought on by SA [39].

Similar results were found in other research, which indicated that a newborn's weight above 3900 g was linked to a higher chance of hypotension. The cause could be because a heavier baby raises the danger of major artery compression from a gravid uterus and the inferior vena cava. Because of the compression, there will be less VR, which lowers preload and puts the patient at risk for hypotension [40].

BSBP was another component that the authors of the study discovered to be linked to hypotension. When comparing SBP of >130 mmHg to BSBP of <120 mmHg, the likelihood of developing hypotension was three and six times, respectively [41].

A related study by Chumpathong et al. likewise showed that 76% of patients who had hypotension (less than 100 mmHg) had the lowest SBP [41]. The patient's BSBP < or = 120 mmHg and height of <155 cm were the parameters associated with an elevated incidence of hypotension [42].

In a different prospective study involving 511 mother-child pairings, with the mothers undergoing SA-mandated elective C-section, authors discovered that a number of risk factors are linked to hypotension, such as age over 35, weight gain, gravidity of ≥4, previous history of hypotension, BSBP of <120 mmHg and HR of >100 beats/min in maternal modelling, fluid preloading, and hypotension, which are twice as likely to occur in people with a BSBP of less than 120 mmHg [43].

The risk of hypotension is strongly correlated with an anesthetist's experience; a study found that when an anesthetist's experience decreased relative to individuals with greater experience, the risk of hypotension increased. This conclusion is highly supported by the earlier Ethiopian findings [44].

A total of 108 pregnant women participated in a cross-sectional study to assess the prevalence and risk factors for severe hypotension during a C-section caused by SA, and the authors discovered that 40.4% of the participants suffered severe hypotension brought on by SA. Additionally, they discovered that parturients who had received an IV preload of crystalloids were shielded from spinal hypotension brought on by SA as opposed to those who had not [45]. Additionally, their study emphasized the use of vasopressors as a key component in the prevention of spinal-induced severe hypotension. Severe hypotension was avoided in the parturient who had co-infusion of ephedrine shortly before SA induction. More than 30% of parturients in Brazil experienced hypotension after giving birth [46,47].

England was likewise observed to have a high incidence, where Crawford found that following the induction of SA for Caesarean delivery, 35.18% of pregnant women develop severe hypotension [48]. Bishop et al. discovered that the prevalence of severe hypotension caused by obstetric spinal injection was 30.4% in South Africa [47]. According to Montoya's 2009 study, 33% of subjects experienced severe hypotension as a result of spinal block [49]. Another study found that 15% of obstetric patients having Caesarean delivery experienced SA-induced severe hypotension [49,50].

Prevention and management

Vasopressors, fluid administration, and posture regimens can all be utilized to increase VR and vascular tone, which are the primary goals of postspinal hypotension prevention and management strategies.

*Vasopressors in SA-Induced Hypotension*

For many years, it was believed that the best vasopressor to use in cases of maternal hypotension was ephedrine, due to its ability to protect uteroplacental blood flow more effectively than α-adrenergic agonists like norepinephrine (NE) and phenylephrine (PE) in experimental investigations. Alpha-adrenergic agonists were then suspected of inducing uterine vascular bed vasoconstriction, which resulted in fetal acidosis. Greiss et al. showed that PE and NE significantly reduced the vasoconstriction of uterine vessels, reducing the impact of high BP on the flow of blood through the uterus, even if SA-induced hypotension was reversed in pregnant sheep [50]. Nevertheless, a larger and more extensive series of clinical trials has shown that PE, NE, and various α-agonists are not only more effective than ephedrine at preventing hypotension but also associated with a lower risk of fetal acidosis. This is because animal experiments do not always translate into replications in human trials [51].

As of now, PE is regarded as the primary vasopressor for preventing hypotension during post-SA. Notably, PE frequently causes a dose-related drop in HR, which is mediated by baroreceptors and a consequent drop in CO. Even at low dosages, this impact can happen even if baseline BP has not increased. Uteroplacental blood flow and maternal CO have a stronger correlation than BP. However, in healthy patients, PE-induced reduction in CO typically has no adverse effects on fetal acid-base balance or Apgar scores, and it is often maintained above baseline levels because of the compensatory increase in CO that occurs after sympathetic nerve blockade. Furthermore, in the majority of patients, the decreased HR may increase back to baseline after stopping PE therapy, and in healthy people, it appears to be insignificant. But in certain situations, like maternal heart disease, preeclampsia, uteroplacental insufficiency, or fetal distress, where the woman and fetus are already impaired, notable changes in HR and CO continue to be concerning [52].

In a double-blinded, randomized study evaluating different set rates of PE infusion, a 20% reduction in maternal CO and HR was observed with 100 μg/min. The current suggestion is to administer an infusion at a rate of 25-50 μg/min, as this was found to be adequate to maintain proper maternal hemodynamics without subjecting the fetus to excessive amounts of PE. While ephedrine is frequently prescribed for the treatment of spinal hypotension, it has potentially harmful effects on fetuses due to the stimulation of fetal metabolism following ephedrine transfer from the placenta and the consequent activation of fetal beta-adrenergic receptors. When 109 patients undergoing elective C-sections at term were compared to high-dose ephedrine and PE infusions, the fetal blood gases improved when the latter was titrated to SBP. Ephedrine had a higher base deficit and a fivefold higher risk of fetal acidosis than PE, according to a recent meta-analysis [53].

The study conducted by Ngan Kee et al. examined the impact of prophylactic PE infusion on preventing hypotension during SA for Caesarean delivery [54]. The findings indicated that PE infusion was superior to a bolus infusion of 100 µg/min in terms of maintaining baseline BP. Furthermore, the incidence of hypotension was lower in the infusion group (23%) as compared to the control group (88%) [54].

According to Prakash et al.'s study, treating hypotension with either 100 µg of PE or 6 mg of ephedrine was beneficial, and the two groups under observation experienced comparable BP reductions [55].

According to a different study, PE (100 µg) infusion on its own was superior to PE and ephedrine combination in controlling the mother's hemodynamics under SA during Caesarean delivery. Research indicates that infusing patients with PE before administering a medication bolus helped them maintain their BP during SA for Caesarean delivery [56,57].

Positioning Regimen: Leg Elevation

The primary cause of postspinal hypotension is decreased vascular tone, which lowers VR and systemic vascular resistance. Postspinal hypotension is mostly caused by reduced vascular tone, which lowers VR and systemic vascular resistance.

Following the spinal block, patients in a randomized control experiment with 150 parturients were placed in a supine position without left uterine displacement. They were then separated into groups for control and leg elevation (LE). Compared to the control group, there was a notable decline in the incidence of hypotension and the need for intraoperative vasopressors in the LE group. LE reduced the incidence of hypotension by 49%. Additionally, it has been reported that the LE group consumed much less ephedrine [58]. On the other hand, the results of Rout et al. revealed no benefit for leg elevation in patients with regard to the incidence of hypotension [57]. Since there were only 31 patients in each group, the authors' study may not have had enough power to support their theory [59]. The incidence of hypotension was observed to be lower in the group treated with a 45° LR in the horizontal plane than in the control group, in contrast to Rout and the other study by Assen et al. [60].

Furthermore, it was demonstrated that the experimental group experienced a significantly lower incidence of hypotension than the group that received a 40° LR in the horizontal plane. This result is in line with the research conducted by Hasanin et al. [57].

Similar to Hasanin and Assen, the incidence of hypotension was reduced in the LR group compared to the control group in the Sari et al. study (p = 0.001) [60]. LR was applied at a 30° angle with the horizontal plane [60,61].

Singh et al.'s research found that raising the lower limb immediately following SA is a useful strategy for preventing hypotension since the rate of hypotension in Group A (leg wrapped) is 10%, while it is 66.66% in Group B (leg unwrapped) [62].

## Conclusions

Hypotension is a common result of SA post-induction and needs to be treated very carefully because it might have negative effects on both the mother and the fetus. A precise measurement of blood volume and an evaluation of fluid response could indicate postspinal hypotension. In the research mentioned above, a high rate of SA-induced hypotension during C-section was observed in various studies. The aforementioned research advises consistently using vasopressors for the prevention and management of SA-induced hypotension. The alpha-agonist was found to be a suitable first-line vasopressor for treating and preventing spinal hypotension since it quickly raises systemic vascular resistance and returns BP to normal.
